# Associations of Quality of Life with Service Satisfaction in Psychotic Patients: A Meta-Analysis

**DOI:** 10.1371/journal.pone.0135267

**Published:** 2015-08-14

**Authors:** Eleni Petkari, Jakob Pietschnig

**Affiliations:** 1 Department of Psychology, Middlesex University Dubai, Dubai, United Arab Emirates; 2 Department of Applied Psychology: Health, Development, Enhancement and Intervention, Faculty of Psychology, University of Vienna, Vienna, Austria; Katholieke Universiteit Leuven, BELGIUM

## Abstract

**Background:**

Quality of life (QoL) has gained increasing attention as a desired outcome of psychosocial treatments targeting psychotic patients. Yet, the relationship between the patients’ satisfaction with services and QoL has not been clearly established, perhaps due to the multidimensionality of the QoL concept and the variability in its assessment.

**Aim:**

This is the first systematic meta-analysis of all available evidence assessing the relationship between QoL and service satisfaction. Methods: In all, 19 studies reporting data of 21 independent samples (*N* = 5,337) were included in the present meta-analysis. In moderator analyses, effects of age, sex, diagnoses (schizophrenia vs. other psychoses), treatment context (inpatients vs. outpatients), study design (cross-sectional vs. longitudinal), and QoL domain (subjective vs. health-related) were examined.

**Results:**

Analyses revealed a highly significant medium-sized effect (*r* = .30, *p* < .001) for the associations of QoL and service satisfaction. Effect sizes were significantly stronger for subjective than health-related quality of life (*r* = .35 vs. *r* = .14, respectively). Moreover, associations with subjective QoL remained largely robust when accounting for moderating variables, although there was a trend of stronger associations for outpatients compared to inpatients. In contrast, effect sizes for health-related QoL were small and only observable for samples with longitudinal designs.

**Conclusion:**

Associations between QoL and service satisfaction appear to be robust but are differentiated in regard to QoL domain. Our findings suggest that agents responsible for service design and implementation need to take the patients’ perception of the service adequacy for achieving QoL enhancement into account.

## Introduction

### 1.1. Disability, mental health services and quality of life

Mental disorders are responsible for approximately 12–15% of the world’s total disability, imposing an enormous cost to patients, families, and society [1] and having a constantly growing impact on the patients’ daily life [2,3]. Consequently, the World Health Organization proposed within the framework of the Quality of Life Project [4] that health and social services should incorporate the enhancement of quality of life in their list of desired outcomes. Specifically, the importance of quality of life improvement in psychotic patients has been receiving increasing attention from psychosocial services, because in more recent years a paradigmatic shift from only treating psychopathological symptoms towards more holistic treatments has taken place. Consequently, research efforts evaluating the effectiveness of psychosocial services by their ability to ameliorate the patients’ quality of life have also increased [5–7].

### 1.2. Quality of Life: A multidimensional construct

The increasing awareness about the importance of patients’ quality of life within treatment setups has led to a substantial increase in research on its potentially meaningful predictors. Varying conceptualizations however, have made it increasingly difficult to obtain one unique and precise definition, perhaps due to the multidimensional nature of the concept. This is of particular importance due to the fact that several quality of life domains have been proposed that appear to be largely independent of each other [8] and which show distinct patterns of associations with other factors such as psychotic symptoms [9]. Such a lack of consensus on the definition of quality of life is reflected by the great variety of instruments that are used for its assessment [10]. Typically, four different conceptualizations are found in the literature, with some authors focusing on objective quality of life which includes the assessment of components such as housing, employment and social functioning [5,11] by using instruments such as the Quality of Life Scale (QLS [12]).

Others emphasise the individuals’ subjective experiences (henceforth: subjective quality of life [13,14]) which are most commonly assessed by the subjective subscales of the Lancashire Quality of Life Profile (LQOLP [15]) or the Manchester Short Assessment of Quality of Life (MANSA [16]), including the assessment of perceived satisfaction with components such as the above mentioned objective indicators (housing, employment, or social functioning). In other conceptualizations and scales, these two domains are incorporated in an overall score [17,18]. Generic test instruments (i.e., combining subjective and objective domains), such as the Quality of Life Instrument (QoLI [19]) or overall scores of the LQOLP and the MANSA are typically used within these assessments. In this context, the WHOQOL Group [4] proposed a more specific approach for quality of life assessment as “*an individual’s perception of his/her position in life in the context of the culture and value systems in which he/she lives*, *and in relation to his/her goals*, *expectations*, *standards and concerns”* [4]. This approach emphasizes the patients’ perspectives and their constant interaction with the environment.

Finally, quality of life relating to the health state and consequences for the patients’ life (henceforth: health-related quality of life [20,21]) has also frequently been described in the literature. This is commonly assessed by the Short Form-36 Questionnaire (SF-36 [22]) or the WHO-Quality of Life Questionnaire (WHOQOL [4,23]). Although health-related quality of life instruments are also based on the patients’ subjective evaluation, their items are focused exclusively on health state (physical and psychological) and the functional impairment that the patient observes as a consequence of such a state, consequently rendering this quality of life component distinct from subjective quality of life.

Due to the plethora of different test measures that are used for quality of life assessment and the multidimensionality of the concept, correlational patterns between these test instruments with other factors (such as sex or social support) seem erratic. This indicates that different domains of quality of life are likely to be differentially associated with other factors such as service satisfaction.

### 1.3. Factors related to Quality of Life

The most commonly proposed moderating variable in the literature is related to psychopathology. However, only a moderate relationship has been reported between reduction of symptom severity and quality of life enhancement, as suggested by a recent meta-analysis [9]. Notwithstanding, psychopathology alone seems to be insufficient to explain the variability in quality of life of patients. Identification of further factors beyond the mere reduction of pathological symptoms appears to be necessary in order to enhance quality of life of psychotic patients, as this may be of particular importance for the practice implementation of the various psychosocial service providers.

### 1.4. Satisfaction with services

One potentially important factor for long-term quality of life that has been frequently emphasized in the literature is effectiveness of healthcare services [24–27]. However, the specific service characteristics that are associated with quality of life improvement are still unclear [28] with one promising candidate having been previously proposed in the form of the patients’ satisfaction with services [29]. Patients’ perception of service characteristics such as adequacy of staff, treatment suitability, as well as quantity and quality of information received (that is, aspects that go beyond the patient’s satisfaction with medication) may play important roles in patient quality of life. Involving the patients’ subjective perspective in the treatment process has been suggested to promote service effectiveness [30–33]. Consistent with these ideas, quality of life improvement has been reported from patients with higher service satisfaction [6].

Similar to the plethora of different quality of life assessment tools, there is a considerable number of different service satisfaction measures, the most widely-used being the Verona Scale of Satisfaction with Services (VSSS [34]) or the Client´s Assessment of Treatment (CAT [35]).

### 1.5. Quality of Life and satisfaction with services

Researchers exploring the relationship between service satisfaction and quality of life have largely reported a positive relationship between the two, but the precise nature of this relationship still remains unclear. Domain differences of quality of life that were assessed (objective vs. subjective vs. overall vs. health-related quality of life), different designs (cross-sectional vs. longitudinal), as well as different treatment contexts (inpatients vs. outpatients; henceforth referred to as treatment context) may account for substantial differences in strength and even direction of observed relationships.

So far, only a few studies have examined the association between service satisfaction and objective quality of life. Whilst one study indicated a positive relationship in a sample of outpatients [36], another two studies did not find such an association for the same treatment context [36] or for inpatients [37]. The majority of the studies have explored the relationship between service satisfaction and the subjective quality of life domain reporting a consistent relationship for outpatients [30,38–41], inpatients [37,42–43], or patients in both treatment contexts [44]. Typically, overall quality of life (i.e., based on composite scores from subjective and objective quality of life domains) has been found to be positively related to service satisfaction [45–49]. The evidence within the literature shows an erratic pattern of strengths of associations between health-related quality of life and service satisfaction depending on treatment context, seemingly indicative of a positive association between them for (i) inpatients [50–52] and (ii) mixed samples [53] (iii) but not for outpatients [54].

### 1.6. The present study

Although most of the available evidence points towards a positive relationship between different domains of quality of life and service satisfaction [52], the strength of associations with different quality of life domains appears to differ considerably and to date still remains unclear.

Evidently, factors affecting treatment outcomes of interventions targeting patients with psychosis are in need of clarification. Therefore, disentangling strength and meaningfulness of associations between service satisfaction and different types of quality of life domains may substantially contribute to tailoring interventions that are likely to enhance treatment effectiveness for psychotic patients. Importantly, identifying variables moderating these relationships may provide useful insights for refining the current treatment approaches of psychosocial service providers. To the knowledge of the authors, there has been no previous meta-analysis exploring within-domain strengths of the relationship between service satisfaction and quality of life in psychotic patients. Presently, we aim to (i) carry out a systematic meta-analysis of all available studies that examine the relationship between quality of life and service satisfaction, (ii) provide an effect estimate for the relationships between single domains of quality of life (subjective vs. health-related) with service satisfaction, and (iii) identify moderating factors of this relationship (i.e., age, sex, diagnosis: schizophrenics vs. other psychoses; study type: longitudinal vs. cross-sectional; treatment context: outpatient vs. inpatient vs. mixed).

## Methods

### 2.1. Literature search

**Fig 1:**
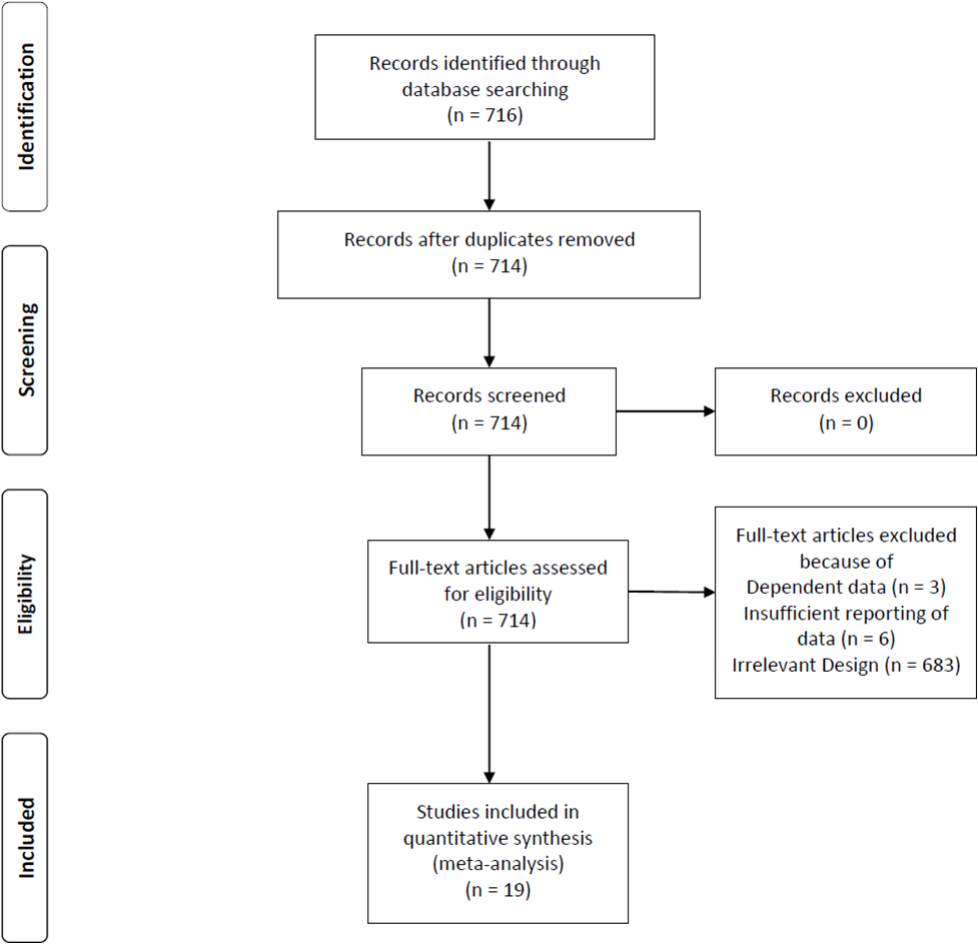
Flow-chart for study inclusion.

In order to identify studies that have explored the relationship between service satisfaction and quality of life, a comprehensive literature search was carried out in five electronic databases (ISI Web of Knowledge, PUBMED, PSYCHINFO, UMI dissertations, Spanish Ministry of Education Theses Database). Search terms were: “‘quality of life’ AND (schizophren* OR psychoti* OR psychosi*) AND ((treatment AND satisfact*) OR (care AND satisfact*) OR (service* AND satisfact*))”, “‘calidad de vida’ AND (esquizofren* OR psicoti* OR psicosi*) AND ((satisfac* AND tratamiento*) OR (satisfac* AND servicio*))”. Identified abstracts of 714 English and Spanish studies were screened for relevance and full texts of potentially relevant studies were obtained. Subsequently, reference lists of full texts were scrutinized for further includable studies (see Fig 1 for a flow-chart of study inclusion). For the preparation of this manuscript, we adhered to the PRISMA [55] guidelines (S1 PRISMA Checklist).

### 2.2. Inclusion criteria

In order for studies to be included in the meta-analysis, the following criteria had to be met: (i) use of a subjective, objective, overall, or health-related quality of life measure for psychotic patients, (ii) assessment of perceived patient service satisfaction including non-pharmacological treatment, (iii) reporting of sufficient statistical parameters to assess bivariate associations, and (iv) independence of data. In cases of data dependencies, preference was given to the larger and most recently reported results.

### 2.3. Coding procedure

Studies that were considered eligible for this meta-analysis were coded independently into categories (i.e., mean sample age; inpatient, outpatient, or mixed sample; length of illness; longitudinal vs. cross-sectional design; percentage of men in samples; patient diagnoses; quality of life domain measured: health-related, objective, subjective, or overall quality of life; year of study publication) by both authors and statistical parameters were recorded (i.e., zero-order correlation coefficients or standardized single regression coefficients, sample sizes). Standardized regression coefficients from primary studies reporting multiple regressions were not included in our meta-analysis due to well-known problems of comparability with zero-order correlations [56] and because the number of potential eligible studies was small (*k* = 1). Discrepancies in coding were resolved by discussion.

For studies that met our inclusion criteria but did not report numerical values for non-significant associations, corresponding study authors were contacted with a request for study details. In cases where study details were not obtainable through personal communications (*k* = 3), effect sizes were fixed to zero following a conservative approach [57].

#### 2.3.1. Quality of life measures

Fourteen of the included studies assessed subjective quality of life by using seven different test instruments. The subjective quality of life subscales of the Manchester Short Assessment of Quality of Life (MANSA [16]) and of the Lancashire Quality of Life Profile (LQOLP [15]) were the most commonly used measures (see Table 1). Ratings of satisfaction with social relationships, financial status, or security are typical examples of item content in such test instruments.

**Table 1:** Details of included samples in the meta-analysis.

Study	*N*	Men (%)	Quality of life measure	Service satisfaction measure	Patient type	Design	*r*
	Health-related quality of life						
Adelufosi et al. (2013)	313	52.1	WHQOL-Bref	Single item measure	outpatients	cross sectional	.00_+_
Lanfredi et al. (2014)	139	64.0	WHQOL-Bref	VSS-54	inpatients	longitudinal	.23
Schmid et al. (2006)	117	71.8	WHQOL-Bref	ZUF-8	inpatients	cross sectional	.27
Wiersma & Van Busschbach (2001)	101	45.5	EuroQOL	VSS-54	mixed	cross sectional	.00_+_
Zendjedjian et al. (2014)	92	51.1	SF-36	Satipsy-22	inpatients	cross sectional	.21
	Subjective quality of life						
Berghofer et al. (2001)	420	53.3	QOLESQ	3 item scale (Berghofer et al.)	mixed	cross sectional	.27
Berghofer et al. (2011)	184	60.9	QOLI	ECS	outpatients	cross sectional	.19
Eklund & Backstrom (2005)	134	61.9	MANSA	2 item scale (Eklund & Backstrom)	outpatients	cross sectional	.44
Eklund (2001)	72	68.1	LQOLP	7 item scale (Eklund et al.)	outpatients	cross sectional	.72
Hansson et al. (2007)	92	46.7	LQOLP	9 item scale (Hansson et al.)	outpatients	longitudinal	.25
Holloway & Carson (1999)	70	65.7	LQOLP	Satisfaction Schedule	outpatients	cross sectional	.21
Petkari (2010)	515	61.0	MANSA	CAT	inpatients	cross sectional	.14
Priebe et al. (1998)	170	55.3	LQOLP	Klientenbogen zur Behandlungsbewertung	inpatients	cross sectional	.39
	90	33.3	LQOLP	Klientenbogen zur Behandlungsbewertung	Inpatients	cross sectional	.00_+_
Priebe et al. (2011)	396	57.3	MANSA	CAT	inpatients	longitudinal	.36
Prot et al. (2011) [82]	81	65.4	SLDS	VSS-54	outpatients	cross sectional	.18
Reininghaus et al. (2011)	708	57.1	LQOLP	PSQ	outpatients	cross sectional	.35
	507	66.3	MANSA	CSQ	outpatients	cross sectional	.45
Rohland et al. (2000) [83]	238	50.0	QOLIMH	8 item scale (Rohland et al.)	outpatients	cross sectional	.50
Ruggeri et al. (2004)	261	37.9	LQOLP	VSS-54	outpatients	cross sectional	.51
Tierney & Kane (2011)	97	44.3	QOLSA	KCSS	outpatients	cross sectional	.42

*Note*:

_+_ = unreported primary effect size that has been fixed to 0

Quality of Life Instruments: WHQOL-Bref = World Health Quality of Life Brief; EuroQOL = Euro Quality of Life; SF-36 = Short Form 36; QOLESQ = Quality of Life Enjoyment and Satisfaction Questionnaire; QOLI = Quality of Life Instrument; MANSA = Manchester Short Assessment of Quality of Life; LQOLP = Lancashire Quality of Life Profile; SLDS = Satisfaction with Life Domains Scale; QOLIMH = Quality of Life Index for Mental Health; Service Satisfaction Instruments: VSS-54 = Verona Service Satisfaction Scale; ZUF = German adaptation of the CSQ-8: Client Satisfaction Questionnaire; ECS = Evaluation of Client Services; CAT = Client Assessment of Treatment; PSQ = Patient Satisfaction Questionnaire; CSQ = Client Satisfaction Questionnaire; KCSS = Kansas Consumer Satisfaction Scale.

In addition, health-related quality of life was assessed in five of the studies using three different measures, the most common being the World Health Quality of Life questionnaire brief version (WHOQOL-Bref [4]). Ratings of patient health status in general and satisfaction with physical or social consequences of the health status are typical examples of item content in such test instruments. No study using objective or overall quality of life test measures met our specified inclusion criteria.

#### 2.3.2. Service satisfaction measures

Sixteen different measures were used for the evaluation of subjective satisfaction with services assessing the patient’s satisfaction with staff, service quality, perception of treatment adequacy, and treatment usefulness. Amongst the included studies, the most commonly used instrument was the Verona Service Satisfaction Scale (VSSS-54 [34]).

### 2.4. Final sample

In all, 19 primary studies (these studies are preceded by an asterisk in the reference list) fulfilled the above criteria and were therefore included in the present meta-analysis. In total there were data from 21 independent samples obtained from 19 included studies. The total number of patients was 5,337, out of which 2,659 were male. In terms of treatment context, 2,935 were outpatients, 1,881 were inpatients, and 521 were of mixed context. All these samples were based on data reporting associations with either subjective or health-related quality of life because no studies reporting objective or overall quality of life fulfilled our inclusion criteria. Sample characteristics are provided in Table 1. All data are provided in the S1 File.

### 2.5. Data analysis

Analyses were performed in five steps. First, weighted effect sizes were calculated according to study precision. Random-effects models were applied in presence of substantial between-study heterogeneity. We provide *I* ^2^ values for all effect size calculations as a descriptive measure of observed heterogeneity. Second, sensitivity analyses were performed. By successively omitting one individual effect size in each turn for overall effect size calculations, potential impact of single atypical observed effect sizes on effect estimations could be assessed.

Third, we applied subgroup analysis to assess differences between the strength of associations of service satisfaction with either subjective or health-related quality of life. In this vein, we expected to observe strength differences because of the previously reported distinct nature of the quality of life domains (see [20]). Furthermore, based on previous findings, differences between effect sizes of cross-sectional and longitudinal studies were expected [58]. Moreover, strengths of correlations between inpatient and outpatient samples were compared in a further analysis for subjective quality of life only.

Fourth, multiple weighted mixed-effects meta-regressions were performed. Originally, age, diagnoses, length of illness, treatment context, and sex were planned to be included in the analysis. However, length of illness needed to be omitted from analyses due to underreporting in primary studies. Studies were weighted according to study precision.

Fifth, influences of potentially confounding publication bias were assessed by means of several methods. All publication bias calculations were based on data of reported effect sizes only. By means of a mixed regression-based method [59], we investigated whether the intercept of a regression line differs significantly from zero when study precision was regressed on effect sizes. Furthermore, we used the Trim-and-fill approach to impute missing studies based on funnel plot asymmetry [60]. Finally, we estimated the number of expected significant results and compared them with observed significant results thus allowing assessment of potential excess of significant results [61].

All procedures were carried out in CMA (Comprehensive Meta-Analysis v2.2.030) and the package metafor [62] in the open-source software environment R 2.12.0 [63]. Effect size estimations were performed by applying Fisher’s *Z* transformation to account for the skewed distributional characteristics of *r*. Reported effect sizes were back-transformed to the *r*-metric for ease of interpretation. Strengths of effect sizes were interpreted according to Cohen’s [64] classification of effect sizes.

## Results

### 3.1. Associations of Quality of Life with service satisfaction

Overall weighted associations of quality of life (i.e., subjective and health-related quality of life; henceforth: combined) and service satisfaction yielded a highly significant medium-sized effect (*r* = .30, *p* < .001; 95% CI [.23, .38]). When limiting inclusion of effect sizes to studies reporting associations with subjective quality of life only, a similar albeit somewhat stronger effect emerged (*r* = .35, *p* < .001; 95% CI [.27, .42]). This association remained robust when examining longitudinal and cross-sectional associations separately (Table 2).

**Table 2:** Associations between satisfaction with services and Quality of Life.

	Overall						Reported effects					
	*k*	*n*	*I* ^2^	*r*	*LCI*	*UCI*	*k*	*n*	*I* ^2^	*r*	*LCI*	*UCI*
All measures	21	4797	86.6	.30_+_ ***	.23	.38	17	3897	82.72	.35_+_ ***	.28	.42
Subjective quality of life	16	4035	84.9	.35_+_ ***	.27	.42	14	3549	87.7	.37_+_ ***	.29	.45
Cross-sectional	14	3547	86.8	.36_+_ ***	.27	.44	13	3457	86.6	.38_+_ ***	.29	.46
Longitudinal	2	488	6.6	.34***	.26	.42	1	92	-	.25*	.05	.43
Health-related quality of life	5	762	64.1	.14*	.01	.26	3	348	<0.1	.24***	.14	.34
Cross-sectional	4	623	65.1	.11_+_	-.03	.25	2	209	<0.1	.24***	.11	.37
Longitudinal	1	139	-	.23**	.07	.38	1	139	-	.23**	.07	.38

*Note*:

_+_ indicates calculation of random-effects models due to between-studies heterogeneity

*I* ^2^ = percentage of variability between effects due to true heterogeneity; *LCI* = Lower bound of 95% confidence interval; *UCI* = Upper bound of 95% confidence interval

* = *p* < .05

** = *p* < .01

*** = *p* < .001

We acknowledge that some readers might feel more comfortable in interpreting overall effect sizes that are based only on coefficients that were reported in primary studies rather than the effect sizes that include unpublished coefficients (i.e., such that have been obtained through personal communications) or coefficients that have been set to zero. Therefore, we provide these estimates in the six right-most columns of Table 2, although we note that these estimates are likely to be inflated due to publication bias.

**Fig 2:**
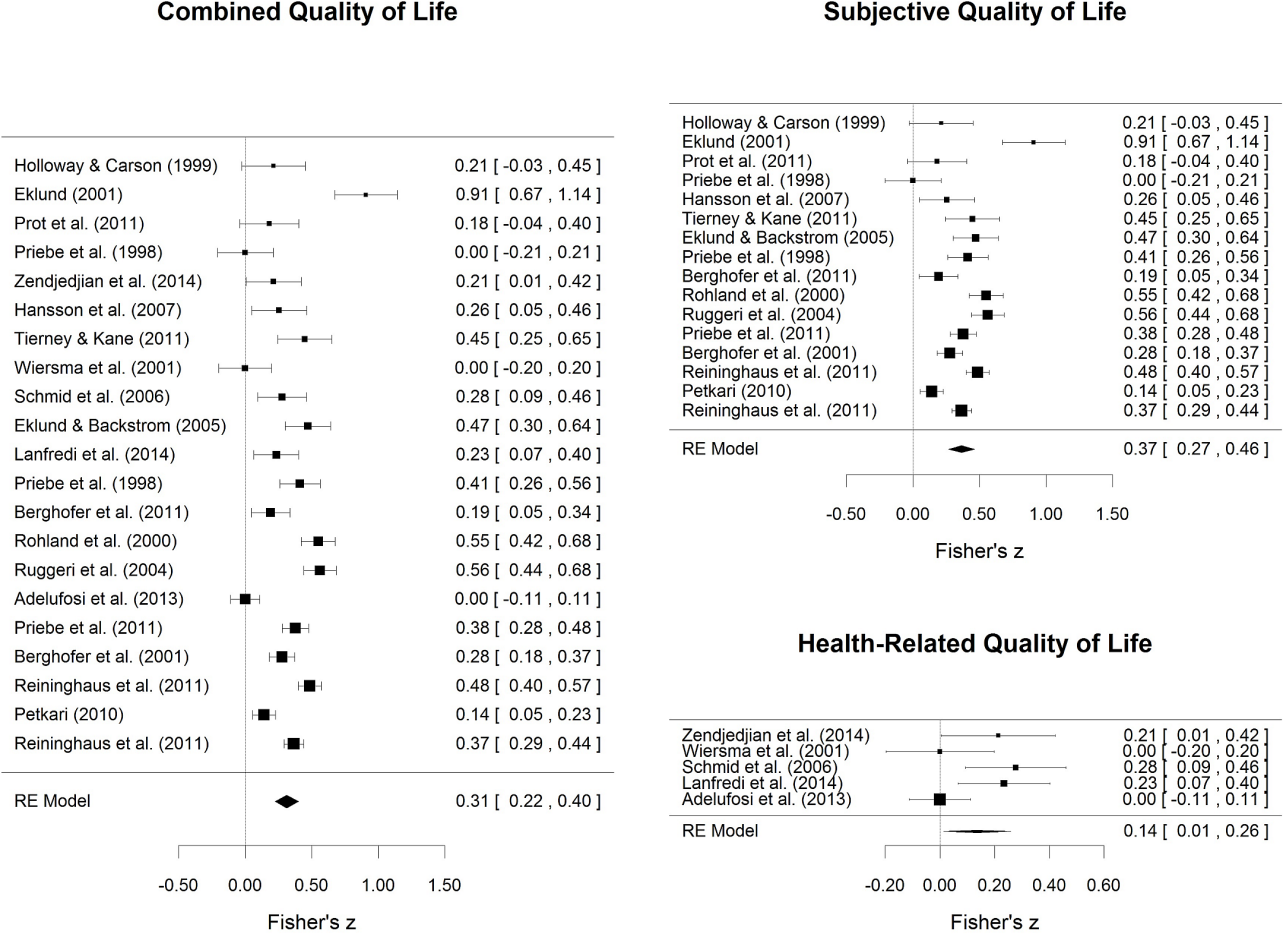
Forest plots for associations of service satisfaction with combined, subjective, and health-related quality of life.

In contrast, the associations of service satisfaction with health-related quality of life were small (*r* = .14, *p* = .03; 95% CI [.01, .26]), showing significant but small longitudinal associations and non-significant cross-sectional associations (Table 2). Effect sizes of combined, subjective, and health-related quality of life with service satisfaction are shown in Fig 2.

Sensitivity analyses showed that when single effect sizes were omitted in each step of overall effect estimation, overall effect sizes remained significant and strengths of associations were only slightly affected for both combined as well as subjective quality of life. This finding indicates robustness of the observed association. For health-related quality of life however, omitting single effect sizes from calculations led in more than half of the effect estimations to a substantial reduction of the effect size in strength and failure to reach significance. This may be on one hand due to the comparatively small number of included coefficients (*k* = 5) but may on the other hand be interpreted as an expression of the weak association between health-related quality of life and service satisfaction.

### 3.2. Subgroup analyses

Subgroup analyses for quality of life domain showed significantly stronger associations of subjective than health-related quality of life with service satisfaction (Cochrane’s *Q* = 8.67, *df* = 1, *p* = .003). These results corroborate robustness of associations of subjective quality of life with service satisfaction as compared to the rather weak association with health-related quality of life, as already evident from sensitivity analyses.

Subgroup analyses for treatment context revealed no significant differences in strength of associations between in- and outpatients for subjective quality of life (Cochrane’s *Q* = 3.03, *df* = 1, *p* = 0.07). However, although nominal significance was not reached, associations for outpatients (*r* = .40) appeared to be considerably stronger than associations for inpatients (*r* = .24). No subgroup analyses were performed for health-related quality of life due to the small number of available data points.

In a supplementary analysis, multiple weighted meta-regressions were calculated using age, diagnosis, treatment context, and sex as predictors. A similar pattern emerged for both combined and subjective quality of life yielding no significant effects for any of the variables (with the exception of higher correlations for outpatients in subjective quality of life). However, findings of the present meta-regressions cannot be considered to be robust because of low study numbers (numerical results omitted for brevity).

### 3.3. Publication bias

None of the four applied methods for detection of publication bias yielded evidence for substantial confounding effect inflation for either combined or subjective quality of life (Table 3). Interestingly though, except for longitudinal studies in subjective quality of life, effect estimates were invariably higher for reported coefficients only than for calculations including all coefficients (i.e., when including coefficients obtained from personal communications with authors of studies that did not report correlation coefficients in published papers; cf. Table 2). This observation illustrates the well-known effect of selective underreporting of non-significant findings in the literature [65]. However, the present effect estimates of reported sample coefficients appear to be comparatively little affected by effect inflation. Due to low sample numbers, no publication bias analyses were calculated for associations with health-related quality of life.

**Table 3:** Tests for detection of publication bias.

		Combined quality of life (*k* = 17)	Subjective quality of life (*k* = 14)
Begg/Mazumdar	*p* value	1.0	.83
Egger	*p* value	.86	.61
Excess significance	χ^2^(1) / *p* value	0.27 / .60	<0.01 / .96
Trim-and-Fill	Observed *r*	.35	.37
	Adjusted *r*	.35	.37
	Added studies	0	0

*Note*: Only published studies were used to calculate measures for publication bias; all calculations were based on random effects models.

## Discussion

Determining the relationship between service satisfaction and quality of life has important implications for the clinical practice, as both variables constitute crucial therapeutic elements for patients suffering from psychotic disorders. To our knowledge, this is the first meta-analysis aiming to provide an estimate for the strength of this relationship whilst considering potential moderating variables. Our results show a robust medium-sized association between combined quality of life and service satisfaction. However, these associations are differentiated in regard to quality of life domain.

Strongest associations were observed for subjective quality of life and service satisfaction. This relationship remained substantial across treatment contexts and study designs. The salience of this relationship is further corroborated by sensitivity analyses which indicate negligible influences of single studies. Publication bias analyses showed only little evidence for effect inflation, thus indicating robustness of results.

On the other hand, our results also show that health-related quality of life and service satisfaction yielded a small association. Moreover, this association was driven by longitudinal studies only, failing to reach significance and yielding an almost trivial effect in cross-sectional designs. However, due to insufficient sample numbers, influences of treatment context could not be evaluated. Sensitivity analyses showed that omission of single studies had considerable influences on strength and significance of the overall effect estimate. Our meta-analysis indicates that subjective and health-related quality of life need indeed to be treated as distinct constructs thus conforming to previous ideas as suggested by Ruggeri and colleagues [8]. In all, associations with subjective quality of life appear to be robustly medium-sized whilst health-related quality of life showed weak associations at best.

### 4.1. Subjective Quality of Life and Service Satisfaction

Subjective quality of life yielded stronger associations with service satisfaction than health-related quality of life, highlighting the well established distinct nature of these quality of life domains [13–14, 20–21]. These differences in the strength of associations emerged consistently for both cross-sectional and longitudinal primary studies. Thus, the results indicate that, at least for subjective quality of life, study design does not appear to play a substantial role for this relationship. The robustness of these associations with subjective quality of life was further corroborated by sensitivity as well as publication bias analyses. Of note, it has been argued that the subjective nature of both service satisfaction and subjective quality of life might be reflective of the same latent variable [39] of subjective appraisal of outcomes. However, recent findings support the distinct nature of the two constructs [66]. This is further corroborated by evidence for discriminant validity in regard to mood [67] as well as the fact that severity of psychotic symptoms shows substantial associations with subjective quality of life evaluations [28] but not with service satisfaction [68].

Thus, although our results confirm that quality of life and service satisfaction share a non-trivial amount of variance, it is suggested that both elements should be included in the assessment of service effectiveness. Both concepts appear to be important for evaluating outcomes of interventions that are tailor-made to the patients’ subjective perception of their own life conditions and received services.

According to previous ideas, one interpretation of our results might be that perceiving good quality of life makes patients happier with the received services [69]. Another interpretation which appears to be more in line with the present findings is that patients’ feelings of being appropriately treated may have both cross sectional and long-term positive effects on their quality of life perception [49].

It needs to be acknowledged that we cannot establish causality in the present examination because we investigated effect sizes based on correlational results only. However, the latter interpretation is consistent with findings of previous studies that provided evidence from longitudinal examinations [26,37,43,49]. Hence, if the treatment environment is perceived as adequate and therefore adjusted to their personal needs, then the patients may perceive a better quality of life. This interpretation is consistent with a proposed subjective quality of life definition reflecting the perceived adjustment between the environment’s characteristics and the patients’ expectations [13].

### 4.2 Health-related Quality of Life and service satisfaction

In contrast, strengths of associations were small for health-related quality of life. Indeed, significant associations were only observable in longitudinal but not cross-sectional studies. Moreover, sensitivity analyses showed that even these observed significant associations would not reach nominal significance in several cases when single studies are omitted, thus indicating the lack of robustness of these associations. According to the definition of the WHO [4], health-related quality of life refers to the health state and its consequences to the person’s living conditions, such as the individual’s possibility to be independent, the medication side effects, or the impact of psychopathology. Those consequences cannot be easily alleviated by treatment interventions especially when it comes to side effects which can have a direct effect on the patient’s quality of life [70], independently of the patients’ perception of the treatment adequacy. However, the patients’ opinion about their treatment may conceivably become important in the long run, as they are able to observe its appropriateness for their health state.

### 4.3. Objective and overall Quality of Life and service satisfaction

It was not possible to calculate the strength of the service satisfaction association with the objective quality of life domain, as insufficient independent primary studies focusing on this quality of life aspect were available. However, no relationship was reported in two out of three studies that explored this association [26,37] but not in Zahid et al. [36]. Therefore, more studies are needed to evaluate how objective life conditions such as housing, financial situation, and size of social network are related to the patients’ service requirements and subsequently perceived satisfaction.

Similarly, no studies investigating quality of life as a composite of subjective and objective domains were eligible for inclusion into our meta-analysis due to our criteria. As reported previously in a number of primary studies using overall quality of life instruments, satisfaction with services was associated with elements evaluating both the patients’ objective reality and its subjective evaluation [45–49].

### 4.4. Moderators

Moderator analyses for subjective quality of life revealed a trend of the treatment context to influence the relationship. Patients receiving services in the community yielded a stronger relationship between level of satisfaction and quality of life perception, as opposed to inpatients. It could be proposed that the stronger relationship reported for outpatients is due to lower levels of psychopathology and thus better illness insight. Subjective appraisals in the context of psychosis might seem inevitably influenced by the patients’ health state. In this case, patients with lower levels of psychopathology would be expected to have a general tendency for more positive subjective appraisals of outcomes and thus would report higher levels of both subjective quality of life and service satisfaction.

However, according to previous findings insight is negatively related to subjective quality of life [70–73] and positively related to service satisfaction [74–75] or not related at all [44, 76]. Therefore, better insight might lead to higher service satisfaction for outpatients but does not seem to account for the stronger association with quality of life reported in our meta-analysis.

In our results, the difference between inpatients and outpatients failed to reach nominal significance. In this vein, it could be argued that patients discharged to the community gradually come to accept the fact that the disease forms part of their lives, and therefore they welcome any intervention that targets the improvement of their life conditions. Subsequently, satisfaction with such interventions would be linked to a better perception of quality of life, as has already been indicated by a number of previous studies [30,38,39,45,66]. Alternatively, this clear trend but failure to reach nominal significance may be due to the inclusion of both hospitalized and long-term institutionalized patients in the inpatient category.

Regarding hospitalized patients, illness-specific factors might intervene in the relationship between service satisfaction and quality of life perception, the most likely candidate being psychotic symptoms. Because these patients find themselves in the acute phase of the disease, the symptoms’ exacerbation plays a crucial role for quality of life perception which has also been supported by findings of Malla and colleagues [77], thus conceivably rendering other factors such as service satisfaction as less influential. Once psychotic symptoms are stabilized and the patients are discharged in the community, they typically engage themselves in the course of rehabilitation processes, where satisfaction with the support of providers may play a more important role for quality of life perceptions.

In contrast, associations in long-term institutionalized patients may conceivably yield a quite different pattern. In fact, their expectations may arguably go beyond the relief of psychopathology only because in long-term patients symptoms might already be alleviated (i.e., due to recession). Such expectations may therefore include the overall satisfaction with the institution as a service system, as this forms part of their everyday life. Consequently, recessive inpatients may be more similar to the outpatients in our meta-analysis regarding their expectations and thus explain the unexpected non-significant difference between treatment contexts.

This idea conforms to the results of Priebe and colleagues [78] who observed that long-term hospitalized patients display improvements in quality of life perception but not in satisfaction with treatment after one year of being discharged into the community. This suggests that once in the community, these recessive patients are different from acute patients, as perhaps other factors beyond service satisfaction such as social relationships [79] become more important for ameliorating quality of life. Obviously, these factors are likely to be different from those that may be important for patients that have been discharged into the community after short-term treatments.

None of the other presently investigated variables, (i.e., sex, age, length of illness, and type of diagnosis) showed meaningful effects on this relationship. However, further variables that were not investigated in the present meta-analysis may conceivably moderate the observed association. For instance, service satisfaction was proposed in the literature as a protective factor buffering the negative influence of other elements, such as negative life events or medication side effects [80] on quality of life.

### 4.5. Limitations

Some limitations need to be taken into account when interpreting our results. First, there has been a great range of measurements used for assessing service satisfaction in the included studies. However, as outlined in our inclusion criteria, great care was taken to only include findings from studies using comparable test instruments and conceptualizations of the variables of interest. Second, it was not possible to conduct moderator analyses for health-related quality of life. However, this was due to the comparatively small number of available effect sizes. Similarly, non-significant results from regression analyses could be due to low numbers of samples in presence of high numbers of predictors. We acknowledge that there may be further potentially moderating variables (e.g., treatment duration) of our observed association which we did not address in the present meta-analysis. However, the number of includable moderators depends on the availability of data from primary studies.

### 4.6. Implications

In all, we could show a robust association between subjective quality of life and service satisfaction. However, associations with health-related quality of life were weak at best. This indicates that service satisfaction may be seen to be indicative of higher appreciation with life as a whole rather than to be limited to the appreciation of health-related aspects or the impact that the disease has on their functioning. These results indicate a need for focusing on patient perceptions of treatments in order to achieve long-term outcomes [81].

The emerging literature on patient-centered care and shared decision making revealed that efforts to enhance patient-centered communication and to promote individuals’ active involvement in mental health treatment decisions lead to significant quality of life improvements. In light of the present results, it seems important for agents responsible for service design and implementation to take the patients’ perception of the service adequacy into account for targeting the amelioration of quality of life.

## Supporting Information

S1 PRISMA Checklist PRISMA checklist. (DOC)

S1 File Primary data of meta-analysis. (XLSX)
